# NeuroVis: Real-Time Neural Information Measurement and Visualization of Embodied Neural Systems

**DOI:** 10.3389/fncir.2021.743101

**Published:** 2021-12-27

**Authors:** Arthicha Srisuchinnawong, Jettanan Homchanthanakul, Poramate Manoonpong

**Affiliations:** ^1^Bio-inspired Robotics and Neural Engineering Laboratory, School of Information Science and Technology, Vidyasirimedhi Institute of Science and Technology, Rayong, Thailand; ^2^Embodied Artificial Intelligence and Neurorobotics Laboratory, SDU Biorobotics, The Mærsk Mc-Kinney Møller Institute, University of Southern Denmark, Odense, Denmark

**Keywords:** visual analytics, information visualization, explainable artificial intelligence, artificial neural networks, embodied neural control, robotics

## Abstract

Understanding the real-time dynamical mechanisms of neural systems remains a significant issue, preventing the development of efficient neural technology and user trust. This is because the mechanisms, involving various neural spatial-temporal ingredients [i.e., neural structure (NS), neural dynamics (ND), neural plasticity (NP), and neural memory (NM)], are too complex to interpret and analyze altogether. While advanced tools have been developed using explainable artificial intelligence (XAI), node-link diagram, topography map, and other visualization techniques, they still fail to monitor and visualize all of these neural ingredients online. Accordingly, we propose here for the first time “NeuroVis,” real-time neural spatial-temporal information measurement and visualization, as a method/tool to measure temporal neural activities and their propagation throughout the network. By using this neural information along with the connection strength and plasticity, NeuroVis can visualize the NS, ND, NM, and NP via i) spatial 2D position and connection, ii) temporal color gradient, iii) connection thickness, and iv) temporal luminous intensity and change of connection thickness, respectively. This study presents three use cases of NeuroVis to evaluate its performance: i) function approximation using a modular neural network with recurrent and feedforward topologies together with supervised learning, ii) robot locomotion control and learning using the same modular network with reinforcement learning, and iii) robot locomotion control and adaptation using another larger-scale adaptive modular neural network. The use cases demonstrate how NeuroVis tracks and analyzes all neural ingredients of various (embodied) neural systems in real-time under the robot operating system (ROS) framework. To this end, it will offer the opportunity to better understand embodied dynamic neural information processes, boost efficient neural technology development, and enhance user trust.

## 1. Introduction

Artificial neural networks (ANNs) have achieved huge success in embodied neural control of robots. One study took inspiration from the neural architecture of *C. elegans* and developed neural architectures for mobile robot parking and robot arm manipulation (Lechner et al., 2019). Other studies equipped multi-legged robots with bio-inspired neural control, which enables the robots to walk or climb on different terrains and adapt to unseen environments (Ijspeert et al., 2007; Arena et al., 2017; Homchanthanakul et al., 2019; Knüsel et al., 2020; Schilling and Cruse, 2020; Thor et al., 2020; Srisuchinnawong et al., 2021; Szadkowski et al., 2021).

**Figure 1:**
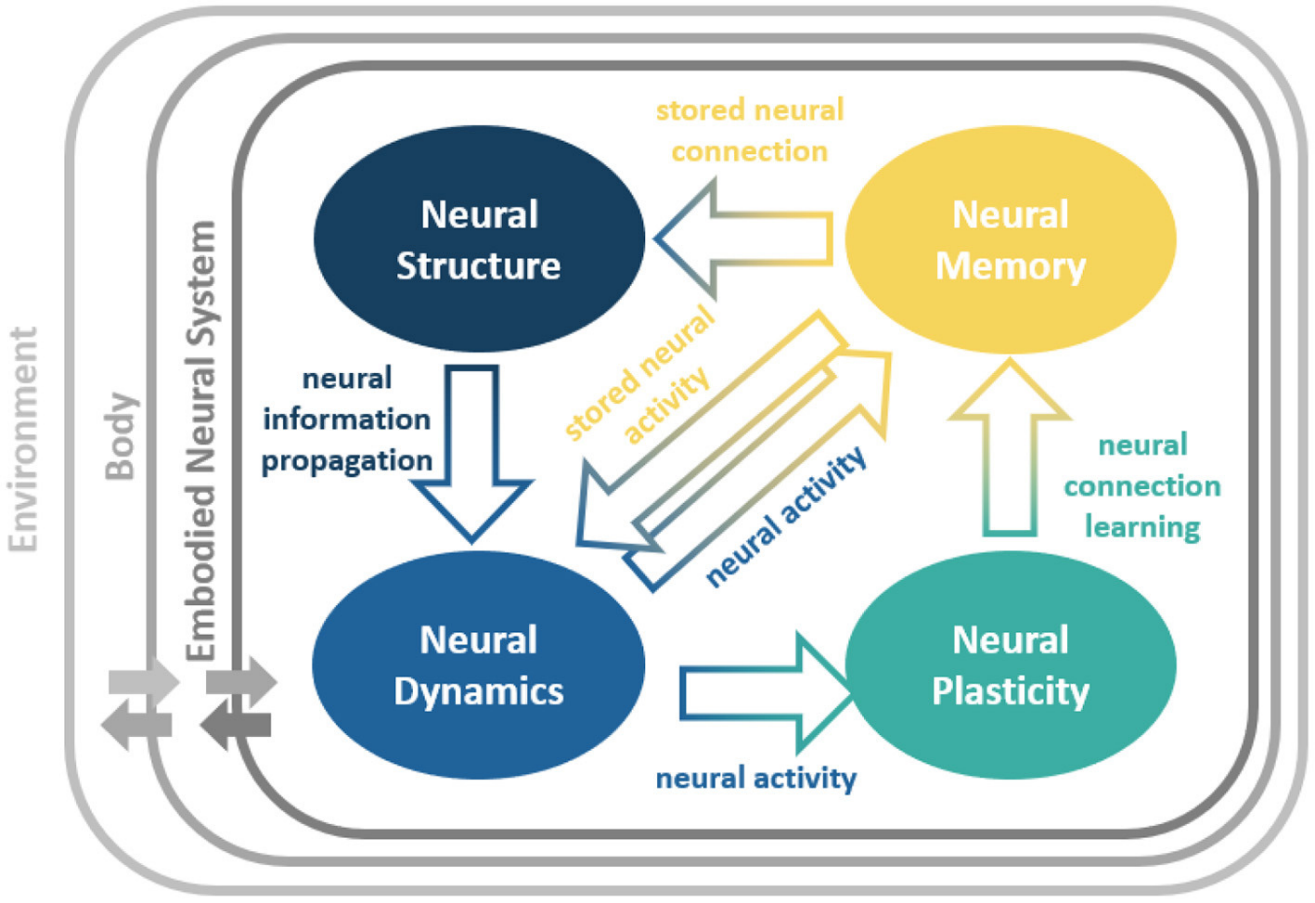
Neural ingredients of an embodied neural system and their relationships. The neural system is embedded into a body and interacts with the environment through the body (Beer, 2008). This complete view describes the complex dynamical neural system-body-environment interactions. NeuroVis offers a possibility to visualize and analyze neural processes of the neural system underlying such complex interactions.

The abilities of such bio-inspired embodied neural systems are the results of the exploitation of four intertwined key neural (spatial-temporal) ingredients (Pau and Johansen, 1990; Rusu et al., 2003; Luque et al., 2014; Cashman et al., 2017; Hohman et al., 2018; Lechner et al., 2019; Rudin, 2019; Shaikh and Manoonpong, 2019; Capolei et al., 2020; Chatzimparmpas et al., 2020; Tang et al., 2020), as illustrated in Figure 1. The neural ingredients are:

- ***Neural Structure (NS)*** which defines the network connections and constrains the information propagation.
- ***Neural Dynamics (ND)*** which alters neural activity patterns according to the propagated neural information.
- ***Neural Plasticity (NP)*** which enables neural connection modification based on the neural activity patterns.
- ***Neural Memory (NM)*** which allows temporary neural information storage (short-term NM) through direct storation of activity patterns or ND and longer-time-scale neural information storage (long-term NM) though connections or NS.

Despite the success that such neural ingredients have produced, the main drawbacks of ANNs are their black box nature and lack of explainability (Rudin, 2019; Chatzimparmpas et al., 2020; Tang et al., 2020). As a consequence, this limits the comprehension to develop efficient embodied neural systems and creates communication barriers between people from different fields (Nordlie and Plesser, 2010; Briscoe, 2012; Ming et al., 2017; Senk et al., 2018).

To address the problem, several explainable artificial intelligence (XAI) techniques have been proposed. One technique is to extract the feature importance level to aid understanding (Tang et al., 2020). However, analysis of the underlying neural mechanisms has not been fully realized. Cloning the original model into a transparent one is an alternative (Ribeiro et al., 2016; Sheh, 2017); however, the cloned model can still differ from the actual one (Rudin, 2019). Therefore, Rudin (2019), Hohman et al. (2018), and Chatzimparmpas et al. (2020) suggested designing interpretable models and analyzing the neural information instead (Hohman et al., 2018; Rudin, 2019; Chatzimparmpas et al., 2020). Even so, interpretable neural models also require effort to understand the ongoing neural processes (Hohman et al., 2018; Chatzimparmpas et al., 2020).

In order to reduce the effort involved and capture the neural processes, graphs and node-link diagrams are extensively used. Graphs suit small time series but not massive whole network data (Lechner et al., 2019; Mehmood et al., 2020; Tang et al., 2020). On the other hand, although node-link diagrams (Pau and Johansen, 1990; Rusu et al., 2003; Cashman et al., 2017; Lechner et al., 2019) suit large-scale analysis, the temporal neural information cannot be tracked online. Thus, combining the advantage of each can lead to effective neural visualization (Chatzimparmpas et al., 2020) where spatial neural information is projected onto the node-link diagram with temporal neural information displayed at nodes and connections.

Examples of those neural visualizations are the topography map (Mehmood et al., 2020) and Neurorobotics Platform (Falotico et al., 2017). They project snapshots of neural activity patterns (partial ND) onto certain positions of the network (NS) without neural activity propagation (ND) and the dynamical changes of neural plasticity (NP) and memory (NM). Another, the Brain Simulator (Simon, 2020), visualizes a neural network as a neuron array with a fixed layout (NS) and discrete connection weights (partial NM and NP) without activity propagation (ND). Other network visualizations demonstrated by Lechner et al. (2019), Manoonpong et al. (2007), and Schilling et al. (2013) display neural networks with ND, but the representations of the temporal neural information of the NP and NM are excluded.

While state-of-the-art neural visualizations may be effective in their own right, their available function remains limited since they cannot visualize all key neural spatial-temporal ingredients (NS, ND, NP, NM) along with their relationship online for real-time embodied neural mechanism analysis (see **Table 1** in the conclusion and discussion section). Furthermore, their application in real-time closed-loop embodied neural control of robots is unsuitable since they typically do not support a practical robot interface, like the Robot Operating System (ROS) (Koubâa, 2019), which effortlessly allows for communication with the most frequently used ROS-based robots in the robotic community. To address this problem, this study presents NeuroVis as a general method/tool to measure and visualize the NS, ND, NP, and NM in real time with the ROS interface for easy NeuroVis-robot communication.

## 2. NeuroVis

NeuroVis^1^ is an open-source real-time neural visualization that measures and visualizes neural information of ANNs. NeuroVis is designed for discrete-time non-spiking neurons (Pau and Johansen, 1990; Rusu et al., 2003; Cashman et al., 2017; Tang et al., 2020; Srisuchinnawong et al., 2021) described by:

(1) $$\begin{array}{r} a_{i}\left[t+1\right]=f\left(\sum w_{ji}\left[t\right]a_{j}\left[t\right]+b\left[t\right]\right) \end{array}$$

*a*_*i*_[*t*] denotes the activity of neuron *i* at timestep *t*. *f* denotes the activation function, *w*_*ji*_[*t*] denotes the weight of the connection from neuron *j* to neuron *i* at timestep *t*. *b*[*t*] denotes the bias of neuron *i* at timestep *t*.

Based on literature reviews, the features of NeuroVis are formulated to fill the existing gaps as follows:

### 2.1. All Four Spatial-Temporal Neural Ingredient Representation

Current methods still do not completely cover NS, ND, NP, and NM. For example, some merely present the activity pattern (partial ND) without information propagation between neurons (Falotico et al., 2017; Mehmood et al., 2020; Simon, 2020). Therefore, one of NeuroVis features is to present all the spatial-temporal neural ingredients using visual attributes with high degree of perceptiveness (e.g., color, intensity, and thickness) to facilitate visual analysis (Alexandre and Tavares, 2010). Specifically, NeuroVis represents NS as a spatial 2D neural position and connection line, based on a node-link diagram, ND as the temporal color gradient of each neuron and connection according to Equations (2, 3), NM as connection thickness according to equation 4, and NP as temporal luminous intensity and changes in connection thickness according to Equation (5).

(2) $$\begin{array}{r} NC_{i}\left[t\right]=clip\left(-1,\gamma_{NC}a_{i}\left[t\right],1\right) \end{array}$$

(3) $$\begin{array}{r} CC_{ij}\left[t\right]=clip\left(-1,\gamma_{CC}a_{i}\left[t\right]w_{ij}\left[t\right],1\right) \end{array}$$

(4) $$\begin{array}{r} CT_{ij}\left[t\right]=|\gamma_{CT}w_{ij}\left[t\right]| \end{array}$$

(5) $$\begin{array}{r} LI_{ij}\left[t\right]=|\gamma_{LI}\left(w_{ij}\left[t\right]\right)-w_{ij}\left[t-1\right]| \end{array}$$

*NC*_*i*_[*t*] denotes the dynamic color gradient of the neuron *i* at timestep *t* (if *NC*_*i*_[*t*] equals to –1, 0, and 1, the neuron will be red, gray, and green, respectively). *CC*_*ij*_[*t*] denotes the dynamic color gradient of the connection from neuron *i* to neuron *j* at timestep *t* (if *CC*_*ij*_[*t*] equals to –1, 0, and 1, the connection will be red, gray, and green, respectively). *CT*_*ij*_[*t*] denotes the thickness of the connection from neuron *i* to neuron *j* at timestep *t*. *LI*_*ij*_[*t*] denotes the luminous intensity of the connection from neuron *i* to neuron *j* at timestep *t*. γ_*NC*_, γ_*CC*_, γ_*CT*_, and γ_*LI*_ denote the scaling factors that scale the neuron color gradient, connection color gradient, connection thickness, and luminous intensity transformation, respectively. *a*_*i*_[*t*] denotes the activity of neuron *i* at timestep *t*. *w*_*ij*_[*t*] denotes the weight of the connection from neuron *i* to neuron *j* at timestep *t*.

### 2.2. General Neural Information Visualization

Due to the inclusion of all NS, ND, NP, and NM, NeuroVis can act as general neural information visualization and substitute other state-of-the-art techniques that do not fully cover all the spatial-temporal neural ingredients (see **Table 1** in the discussion and conclusion section for the summary). From this point of view, the neural information measurement and visualization of NeuroVis can correlate to the results of other techniques. To demonstrate this, a use case (described below) presents the example of interpretation using NeuroVis along with references to other works that employed different techniques (e.g., cross-correlation and mutual information analysis) but reported similar results.

### 2.3. Straightforward Interpretation

NeuroVis makes two improvements from the existing methods (Manoonpong et al., 2007; Schilling et al., 2013; Lechner et al., 2019; Simon, 2020) in order to straightforwardly provide the overview and dynamics of (embodied) neural systems and allow non-experts to easily understand and interpret the neural visualization of NeuroVis.

Firstly, in contrast to other visualization methods (Manoonpong et al., 2007; Schilling et al., 2013; Lechner et al., 2019; Simon, 2020) that are based on static symbol and color representations of connection types^2^, Neurovis encodes neural activity and synaptic temporal dynamics into dynamic color gradients of connections according to Equation (3).

**Figure 2:**
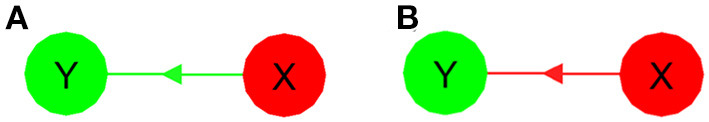
Example of a neural network with two neurons (i.e., neuron X with negative activity and neuron Y with positive activity) and the inhibitory (negative) connection between them. **(A)** Visualization of NeuroVis. NeuroVis automatically converts a combination of the negative activity from X and the negative connection into the visible actual input (i.e., here positive (excitatory) input) to Y. **(B)** Visualization of the other methods. It does not automatically convert the combination as such the actual input to Y is not visible.

Using this approach, one has to simply consider only the input to each neuron in order to analyze why the neuron is active instead of considering both the connection type and presynaptic neuron's activity as usually required by the other methods. Taking Figure 2A as an example of the visualization obtained from NeuroVis, Y receives a positive signal (green) and becomes positive (green). In contrast to this, when employing the other methods with static symbol and color representations, both the presynaptic neuron's activity and connection weight or type must be taken into account. Taking Figure 2B as an example of the visualization obtained from the other methods, Y becomes positive (green) since X is negative (red) and the connection weight is negative (red).

Secondly, while the other neural visualizations (Manoonpong et al., 2007; Schilling et al., 2013; Lechner et al., 2019) do not display the NP (dynamically changing connections), Neurovis displays connection plasticity through connection thickness (according to Equation 4) and also highlights the change of the plasticity with a luminous yellow outline (according to Equation 5), as shown in **Figure 4A**.

### 2.4. Practical ROS Interface

**Figure 3:**
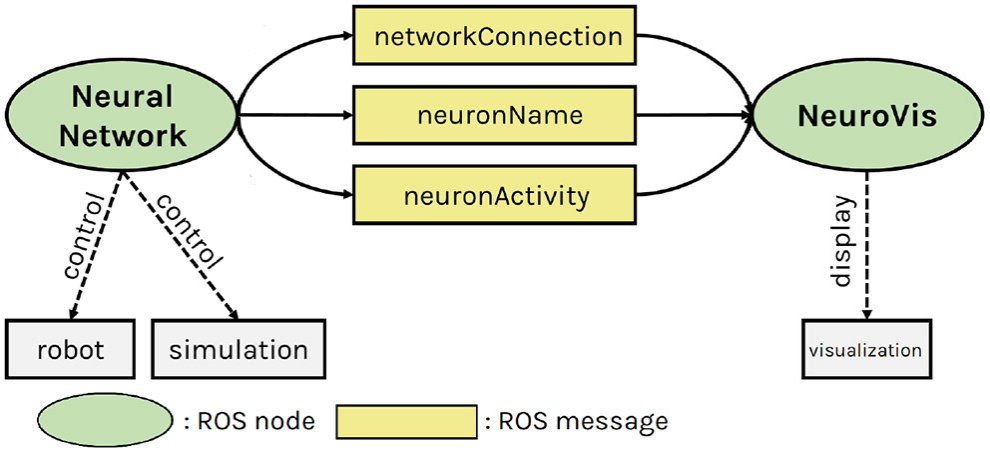
The NeuroVis-ROS interface. Neurovis ROS node creates a visualization using three ROS messages (including network connection, neuron name, and neuron activity) from another ROS node implementing the neural network that interfaces with a real robot or simulation.

ROS (Koubâa, 2019) is a framework that allows communication between multiple ROS nodes locating on either the same or different devices. Under this framework, NeuroVis acts as a ROS node that subscribes to those real-time (≥ 20*Hz*) messages and monitors embodied neural robot control during robot operation, as shown in Figure 3. Since NeuroVis operates under ROS, neural data can be recorded using a ROS package called rosbag. NeuroVis also has a function to automatically record the NeuroVis visualization as a video file (see also the git repository of NeuroVis^1^).

## 3. NeuroVis Use Cases

This section presents three use cases in which NeuroVis was used to visualize and interpret three different (embodied) neural networks. The first use case aims to provide the design principles and demonstrate that NeuroVis covers all four neural ingredients (the first feature; subsection 2.1). Besides it evidences the opportunity to substitute other state-of-the-art information visualization techniques with this method (the second feature; subsection 2.2) and provides an example of interpreting the neural network (the third feature; subsection 2.3). The second use case aims to evaluate the NeuroVis-ROS interface on a small-scale embodied neural network that was learned online using reinforcement learning for hexapod locomotion generation (the fourth feature; subsection 2.4). Lastly, the third use case aims to further evaluate the NeuroVis-ROS interface on a larger-scale embodied neural network (the fourth feature; subsection 2.4) and demonstrate an example of interpreting a complex embodied neural network (the third feature; subsection 2.3).

### 3.1. Use Case 1: Function Approximation

**Figure 4:**
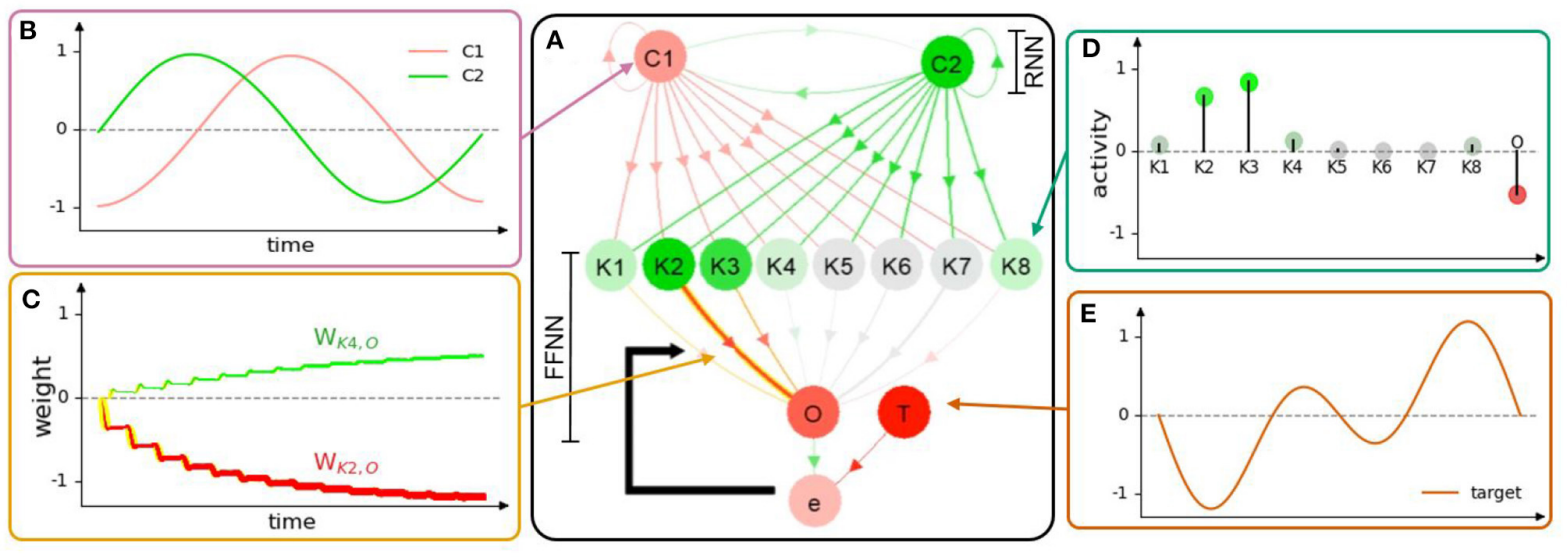
**(A)** Neural visualization of the network in the first use case. **(B)** C1 and C2 activities or outputs of the RNN. **(C)** Connection weights of K2 and K4 to O, where the change of line thickness and luminous intensity (yellow) represents the weight changes (NP). Red and green colors indicate the propagated signals that contribute to the decrease and increase of activity, respectively. **(D)** The activities of K1-8 and O are mapped according to the color of the gradient. The saturated green node, saturated red node, and color-less node indicate highly positive, highly negative, and inactive (≈0) neural activities, respectively. **(E)** The target function at neuron T of the first use case. Note that the graphs **(B–D)** illustrate the neural information analysis based on the visualization of NeuroVis. In this study, for simplicity, only the weights between K1-8 and O are adapted online to observe the NP and NM. A video of this use case can be seen at Supplementary Video 1^3^.

The first use case involves a modular neural network with recurrent and feed forward topologies as an example as shown in Figure 4A. This architecture has been used to produce an arbitrary periodic signal for robot locomotion (Thor et al., 2020; Srisuchinnawong et al., 2021). The dynamics of the network are governed by:

(6) $$\begin{array}{r} C_{1}\left[t+1\right]=tanh\left(w_{11}C_{1}\left[t\right]+w_{21}C_{2}\left[t\right]\right) \end{array}$$

(7) $$\begin{array}{r} C_{2}\left[t+1\right]=tanh\left(w_{12}C_{1}\left[t\right]+w_{22}C_{2}\left[t\right]\right) \end{array}$$

(8) $$\begin{array}{r} K_{i}\left[t\right]=gaussian\left(C_{1}\left[t\right],C_{2}\left[t\right]\right)=exp\left(-\sigma \overset{2}{\underset{i=1}{\sum }}{\left(C_{i}\left[t\right]-\mu_{i}\right)}^{2}\right) \end{array}$$

(9) $$\begin{array}{r} O\left[t\right]=\overset{k}{\underset{i=1}{\sum }}w_{K_{i}O}K_{i}\left[t\right] \end{array}$$

*C*_*i*_[*t*], *K*_*i*_[*t*], and *O*[*t*] denote the activities of the neuron Ci, Ki, and O at timestep *t*, respectively. *w*_*ij*_ denotes the weight of the connection from neuron *i* to neuron *j*. *k* denotes the number of the kernel. Here, eight kernels are used. *tanh*(), *gaussian*(), and *exp*() denote hyperbolic tangent activation function, Gaussian activation function, and exponential functions, respectively. σ and μ_*i*_ denote the parameters of the Gaussian function, which are set to 40 and eight interpolated points between –1 and 1, respectively.

In this use case, *w*_11_, *w*_21_, *w*_12_, and *w*_22_ are predefined and fixed as 1.000, 0.049, -0.049, and 1.000, respectively (Pasemann et al., 2003). On the other hand, the connections between K1-8 and O were trained online to approximate two nonlinear target functions (at neuron T) with supervised learning according to:

(10) $$\begin{array}{r} w_{K_{i}O}\left[t+1\right]=w_{K_{i}O}\left[t\right]+\eta K_{i}\left[t\right]\left(T\left[t\right]-O\left[t\right]\right) \end{array}$$

*w*_*K*_*i*_*O*_[*t*] denotes the weight of the connection from the kernel neuron Ki to the output neuron O at timestep *t*. η denotes the learning rate, which is 0.01 in this case. *K*_*i*_[*t*], *T*[*t*], and *O*[*t*] denote the activities of the kernel neuron Ki, the target T, and the output O at timestep *t*, respectively.

During the training process, NeuroVis displayed the visualization at the display frequency of 40 frames per second (40 Hz) under the ROS interface (see Supplementary Video 1^3^ for the demonstration). Note that the modular network is considered here to show that NeuroVis can be applied to both discrete-time non-spiking recurrent and feed forward networks. The results are described below alongside the network interpretation and the structural elements from which they mainly derive since this better reflects the tight intertwining of the neural ingredients and NeuroVis functions.

#### 3.1.1. Neural Structure

The NS determines whether each pair of neurons connects or not. It represents the spatial neural information structure of the network. The network connection is mathematically modeled as a Boolean connection matrix (*C*) (Capriglione et al., 2016):

(11) $$\begin{array}{r} C=\left[\begin{array}{ccc} c_{11} & \ldots & c_{1m}\\ \vdots & \ddots & \vdots \\ c_{n1} & \ldots & c_{nm} \end{array}\right] \end{array}$$

where the element at row i column j (*c*_*ij*_) denotes the existence of the connection from neuron i to neuron j.

NeuroVis thus draws a node-link diagram according to the connection matrix, as depicted in Figure 4A. Connections are drawn to link pairs of neurons to identify their existence. The neurons are simulated at adjustable positions (Alexandre and Tavares, 2010) so that the layout follows the explanation order (Rusu et al., 2003; Lechner et al., 2019). For example, in this use case (Figure 4A), the neurons are positioned according to the functions of the modular network. The upper part of the network layout is a two-neuron recurrent network (RNN), namely, C1 and C2, with a hyperbolic tangent activation function. The RNN generates basic sinusoidal-like signals which are propagated downward to the lower part of the layout which is a feed-forward neural network (FFNN) with eight hidden neurons (K1-8) and one output neuron (O) for periodic signal shaping. However, we can also position neurons in relation to the robot system that they control (see the third use case).

NeuroVis displays the connections with arrows. One can observe which inputs are taken by a certain neuron and to which neuron the output transmits. The RNN with the predefined weights produces two sinusoidal-like signals (i.e., activities of C1 and C2, Figure 4B), which are transmitted to K1-8 of the FFNN. By doing so, the sinusoidal-like signals are shaped by the Gaussian activation function with different parameters. The activities of K1-8 are then weighted and summed to produce the output O with respect to a target function T (Figure 4E). The error e is then calculated as e = T − O. Finally, the network takes e to adapt the weights between K1-8 and O online using a standard delta rule, as shown in Equation (10). The ND, NP, and NM of the network described below are constrained by this NS.

#### 3.1.2. Neural Dynamics

**Figure 5:**
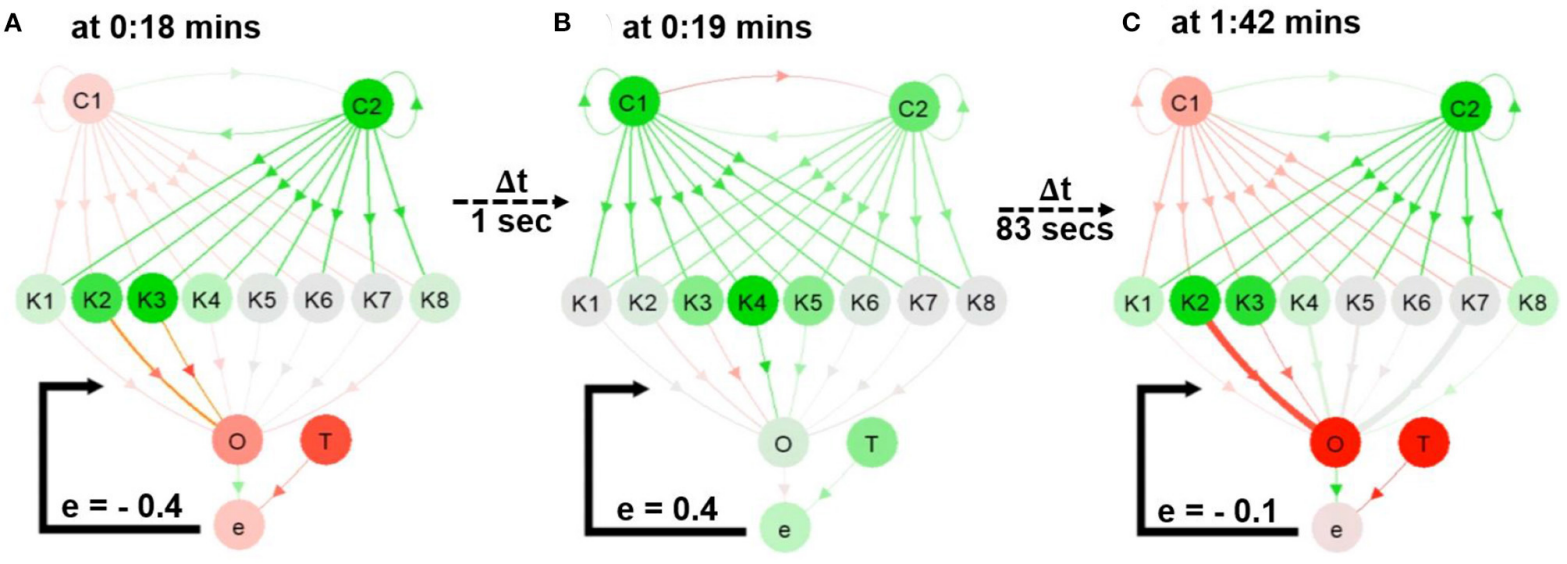
Snapshots from the first video ^3^. **(A,B)** The network during online learning showing the changes in neural activities and connection weights. **(C)** The weights converging to different thicknesses in the network. A video of this use case can be seen at Supplementary Video 1^3^.

The ND represents temporal neural information which describes the evolution of the activity pattern according to the information propagated (see Figure 5). Since neurons are usually analyzed as negative, inactive, or positive (Lechner et al., 2019), NeuroVis scales the measured neuron activities to values between –1 (negative activity; red) and +1 (positive activity; green), where 0 denotes inactivity (gray). It then presents neural activity using colors and gradients (Alexandre and Tavares, 2010) (see Figure 4D). The information propagated through a connection is also typically interpreted as the signal, contributing to the increase or decrease in activity (Lechner et al., 2019). Thus, NeuroVis multiplies the activity using the connection weight to obtain the hue and gradient of the connection, representing the information transmitted through such connection (Figures 4A,C).

By observing at C1 and C2 through NeuroVis in Supplementary Video 1 and in Figure 5, we can track and analyze the ND of the network as follows. At 0:18 min (Figure 5A), C2 has a strong positive activity (green). It propagates the activity directly to C1 via the excitatory connection (green), making C1 become positively active (green) at 0:19 min (Figure 5B). Afterward, C1 turns highly positive (green), transmitting its activity to C2 via the inhibitory connection (red) and inhibiting the C2 activity (to become less green, see Figures 5A,B). This ND of the RNN makes C1 and C2 change from red to green repeatedly and vice versa.

Concurrently, the oscillating activities of C1 and C2 propagate to K1-8. Due to the Gaussian activation function with different centers, K1-8 can be observed to receive the same inputs but exhibits different activities, and K1-8 becomes active at certain input patterns. For example, in Figures 5A,C, whenever C1 is pale red, and C2 is bright green, K1-4 and K8 are active. As a result of such ND, a group of three to five neurons is activated, producing the ripple from the left to the right.

If we compare NeuroVis to cross-correlation (Lechner et al., 2019) and mutual information (Shwartz-Ziv and Tishby, 2017) analysis, which are the typical methods for analyzing neural activity patterns, NeuroVis can also be used to analyze the neural activity patterns in a similar way. This is due to the fact that cross-correlation and mutual information (Equations 12–14) are both proportional to the number of times when such patterns occurs (*n*(*x*) and *n*(*x, y*)).

(12) $$\begin{array}{r} CC\left(x,y\right)=\underset{x}{\sum }\underset{y}{\sum }xyp\left(x\right)p\left(y\right) \end{array}$$

(13) $$\begin{array}{r} I\left(X;Y\right)=\underset{x}{\sum }\underset{y}{\sum }p\left(x,y\right)log\frac{p\left(x,y\right)}{p\left(x\right)p\left(y\right)} \end{array}$$

(14) $$\begin{array}{r} p\left(x\right)=\frac{n\left(x\right)}{\sum_{x}n\left(x\right)}\;and\;p\left(x,y\right)=\frac{n\left(x,y\right)}{\sum_{x}\sum_{y}n\left(x,y\right)} \end{array}$$

*X* and *Y* denote the discretized activity patterns, *x* ∈ *X* and *y* ∈ *Y*. *CC*(*x, y*) and *I*(*X*; *Y*) denote the cross-correlation value when event *x* and *y* occur and mutual information between the two variables, respectively. *n*(*x*) denotes the number of times when *x* occurs. *n*(*x, y*) denotes the number of times when both *x* and *y* occur.

**Figure 6:**
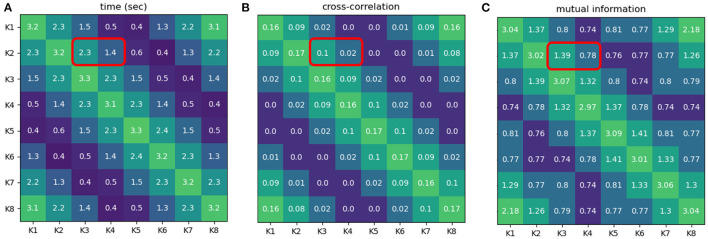
**(A)** Time intervals when pairs of neurons displayed in NeuroVis become active together. **(B)** Correlation values when pairs of neurons activate [*CC*(*K*_*i*_[*t*] > 0.05, *K*_*j*_[*t*] > 0.05)]. **(C)** Mutual information between pairs of neurons [*I*(*K*_*i*_, *K*_*j*_)]. The greenish color indicates a longer time interval where two neurons activate together, higher mutual information value, and higher correlation value, while the blueish color indicates a shorter time interval where two neurons activate together, lower mutual information value, and lower cross-correlation value. Note that a neuron is considered active when its absolute activity is above 0.05. A video of this use case can be seen at Supplementary Video 1^1^.

According to the result obtaining from the demonstration in this experiment (see Figure 6 and the Supplementary Video 1^3^), the time intervals of neural activities (from NeuroVis) can be also used to analyze the neural activity patterns since it has the same trend as the correlation values from Equation (12) and mutual information from Equation (13). For example, the time interval when K2 and K3 activate together is greater than when K2 and K4 activate together (2.3 and 1.4 s, respectively, see the red squares in Figure 6A). This is comparable to the cross-correlation analysis demonstrating that the correlation value of K2 and K3 is also greater than that of K2 and K4 (0.1 and 0.02, respectively, see the red squares in Figure 6B). In addition, with mutual information analysis, the mutual information between K2 and K3 is greater than that of K2 and K4 (1.39 and 0.78, respectively, see the red squares in Figure 6C).

#### 3.1.3. Neural Plasticity

The NP represents neural temporal information and enables connection weight adaptation according to the ND, leading to long-term NM. Accordingly, NeuroVis employs the update of connection weight to track NP (Chatzimparmpas et al., 2020). The NP is usually considered to be quantitative data (Cashman et al., 2017; Ming et al., 2017), indicating the degree of weight change. NeuroVis thus presents weight updates indicated by Equation (10) using time evolution of the connection size/thickness and highlighting it with a luminous yellow outline (Alexandre and Tavares, 2010).

As depicted in Figure 4C and in Supplementary Video 1^3^, when a connection weight becomes more positive or negative, the thickness enlarges and casts a luminous yellow glow. At 0:18 min (Figure 5A), a significant error between O and T exists (i.e., –0.4). As a result, the connections from K2 to O and K3 to O cast a luminous glow as they are being updated/thickened. Later, at 1:42 min (Figure 5C), the error between O and T reduces to -0.1, and the weights converge. This is indicated by a lack of luminous outline, representing no weight change. Besides, the connection from K2 to O changes from a thin red (Figure 5A) to thick red connection (Figure 5C), indicating a change from a small negative to a large negative (inhibitory) weight. This demonstrates that NeuroVis can visualize and analyze temporal and maximal weight updates over time (Cashman et al., 2017; Ming et al., 2017).

#### 3.1.4. Neural Memory

The NM consists of long-term NM and short-term NM.

##### 3.1.4.1. Long-Term NM

**Figure 7:**
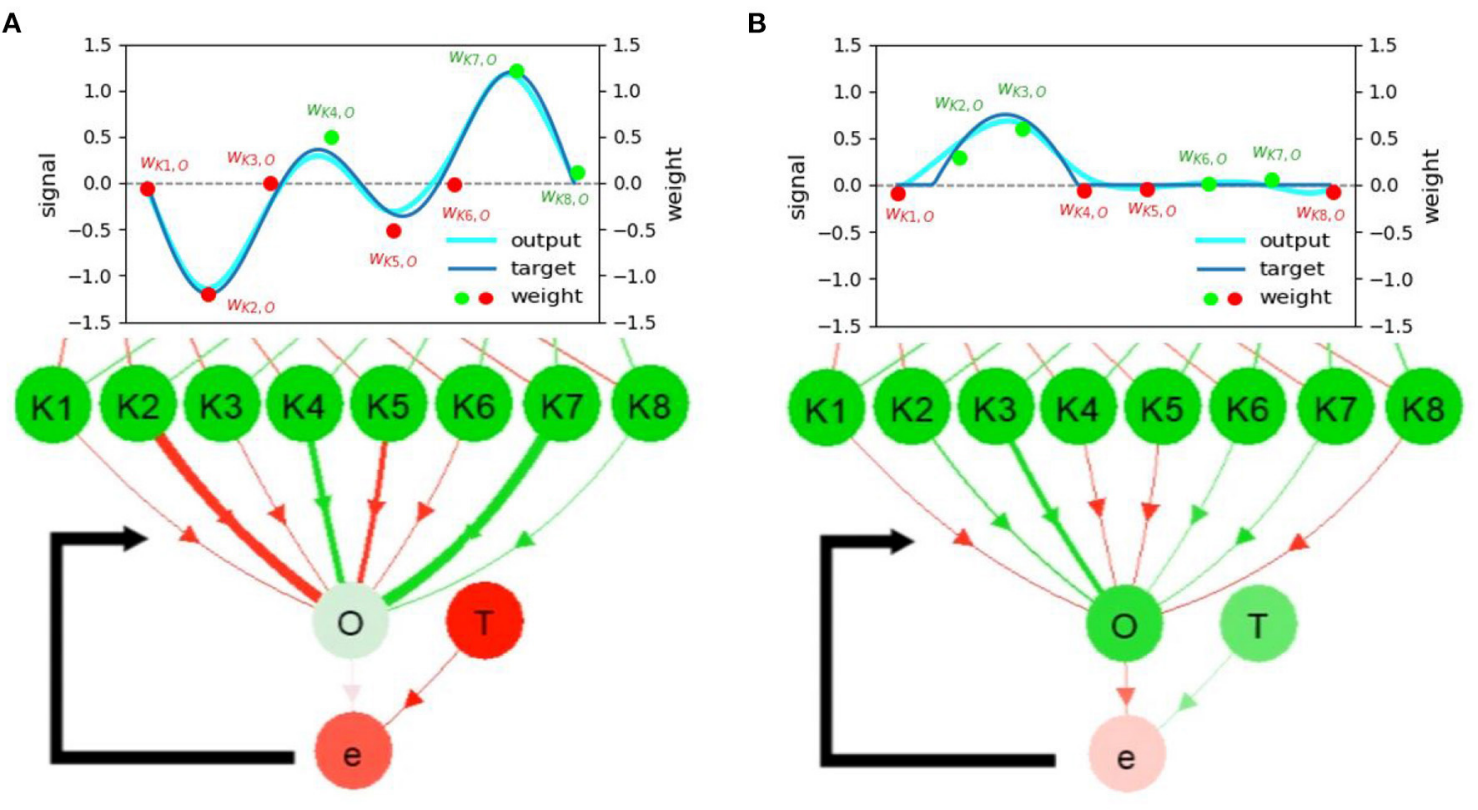
Snapshots of the networks trained with **(A)** the first and **(B)** the second target functions. Snapshots are from Supplementary Video 1^3^ after the training end. The relationship between the converged weights, output, target, and neural visualization is depicted. Scatter plots and their color represent the weight magnitude and the sign of information transmitted through the connections, respectively. Note that *w*_*i,j*_ denotes the connection weight from neuron *i* to neuron *j*. A video of this use case can be seen at Supplementary Video 1^1^.

Long-term NM is the ability to store certain neural information as connection weight. Since connection weight is considered quantitative data, being proportional to the amount of information that one neuron propagates to another, NeuroVis maps the measured connection weight value to connection thickness (Alexandre and Tavares, 2010) as depicted in Figure 7. With this principle, large/thick connections are those with strong influence. For instance, in Figure 5C or Supplementary Video 1^3^ at 1:42 min, the path from C2 to K2 and O mainly contributes to the network output since it is the thickest/largest. Hence, this example shows that NeuroVis can be used to simply monitor and analyze NM instead of looking at only the weight values as usually done (Pau and Johansen, 1990).

Apart from this, NeuroVis also provides insight into how the ND in time space is memorized in weight space. Considering Figure 7 along with Supplementary Video 1^3^ after 1:40 min, NeuroVis shows that the converged weights are the direct representation of the output/target for this neural network. The outputs/targets are encoded as the connections with various thicknesses as shown in Figure 7. Different thicknesses are also mapped to their corresponding bars (in Figures 7A,B) to present the relationship between the neural visualization and graph.

##### 3.1.4.2. Short-Term NM

**Figure 8:**
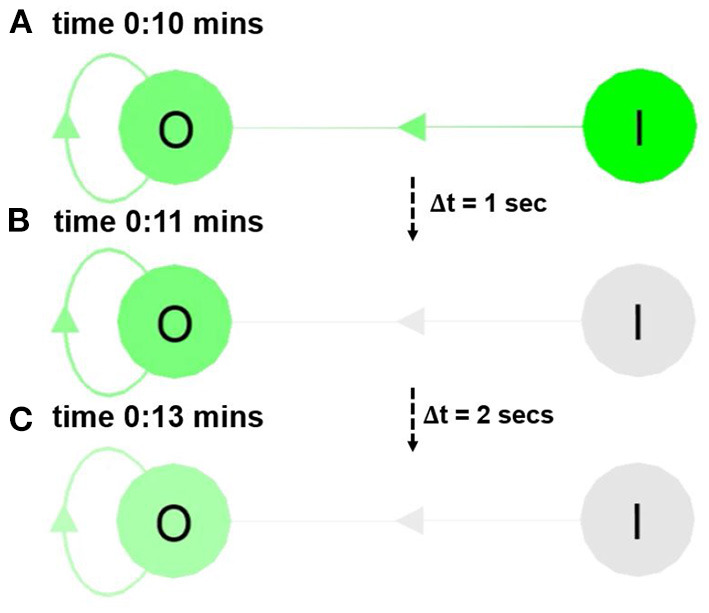
Snapshots from Supplementary Video 2^4^ showing the neural visualization of the ANN with two neurons (i.e., I and O). **(A)** At 0:10 min, the activity of I transmits to O, causing O to become active (turn green). **(B,C)** At 0:11 and 0:13 min, O remains active due to its recurrent connection. This shows the ND as the short-term NM. A video of this use case can be seen at Supplementary Video 2^4^.

Short-term NM is the ability of the ND to retain neural activity for a certain period of time, which can be visualized via the neural color gradient of NeuroVis (in Figure 8 and Supplementary Video 2^4^. The video is available at www.manoonpong.com/NeuroVis/video2.mp4). At 0:10 min, the activity of neuron I transmits to neuron O. As a result, O becomes active and turns green. Later, from 0:11 to 0:13 min, the ND of O with the recurrent connection provides the recurrent input that prolongs the activity even if the input signal from I has vanished.

### 3.2. Use Case 2: Robot Locomotion Control and Learning

**Figure 9:**
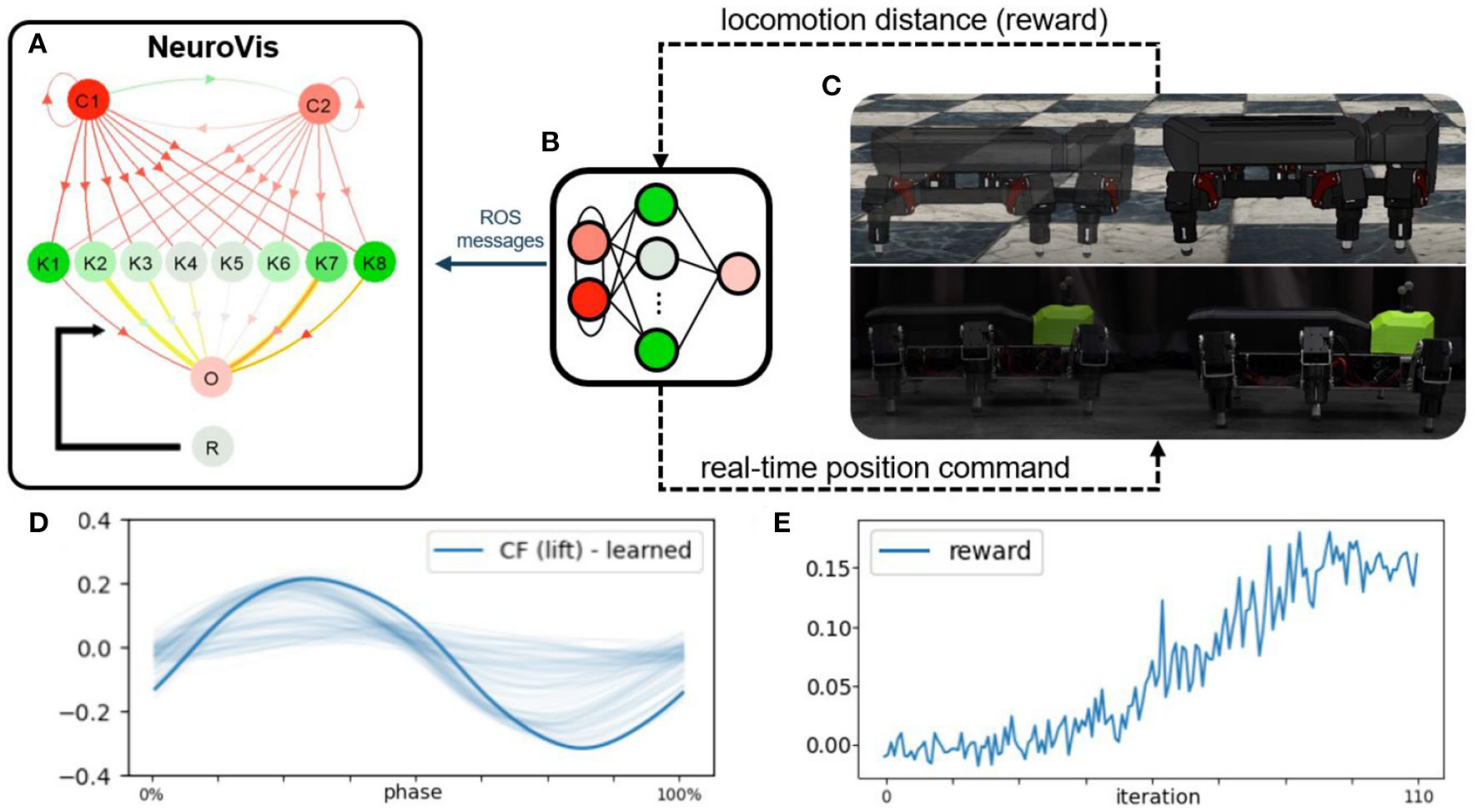
The second use case showing the use of NeuroVis for robot locomotion control and reinforcement learning in a closed-loop embodied neural network. **(A)** Neural visualization of NeuroVis. **(B)** Neural control that is used in the second use case and visualized in **(A)**. **(C)** Simulated and physical robots. **(D)** Learned optimal output signal for optimal swing and stance patterns. **(E)** Example of the reward signal for learning the connection weights between K1-8 and O to obtain the optimal output signal. A video of this use case can be seen at Supplementary Video 3^5^.

In Supplementary Videos 3^5^, the second use case demonstrates the NeuroVis-ROS interface to monitor and analyze an embodied neural locomotion control implemented on a hexapod robot (Figure 9) (Thor and Manoonpong, 2019; Thor et al., 2020). The neural architecture used in the first use case is still employed here, but its weights are optimized with reinforcement learning (i.e., advantage policy gradient Sutton et al., 1999) to automatically generate robot locomotion with optimal swing and stance patterns under a fast tripod gait. The learning objective was to let the robot learn to speedily walk forward, with forward displacement acting as a reward function for adapting the connection weights between K1-8 and O (see Equation 15).

(15) $$\begin{array}{l} w_{K_{i}O}\left[b+1\right]=w_{K_{i}O}\left[b\right]\\ \;+\eta \left(r\left[b\right]-r_{avg}\left[b\right]\right)\underset{t}{\sum }\pi \left[t\right]K_{i}\left[t\right]\left(\pi \left[t\right]-O\left[t\right]\right) \end{array}$$

*b* denotes the number of training iteration. *t* denotes the number of timestep in one training iteration. *w*_*K*_*i*_*O*_[*b*] denotes the weight of the connection from the kernel neuron Ki to the output neuron O at training iteration *b*. *r*[*b*] denotes the distance that the robot walks in iteration *b*. *r*_*avg*_[*b*] denotes the moving average of such. η denotes the learning rate which is set to 0.01. π[*t*] denotes the stochastic control policy under normal distribution (*O*[*t*] + *noise*) at timestep *t*. *K*_*i*_[*t*] and *O*[*t*] denote the activities of Ki and O at timestep *t*.

After the learning, the network was transferred to a physical hexapod robot. During all these processes, the controller (Figure 9B) published all the neural information via ROS messages to NeuroVis, which created the neural visualization accordingly (Figure 9A). With this use case, NeuroVis displayed the neural visualization of the small-scale neural control with 12 neurons and 28 connections at a frequency of 40 frames per second under the ROS interface. According to Supplementary Video 3^5^, one can observe the NS, activity pattern and signal propagation (ND), the evolution of connection weights (NP), and how the ND of the output is memorized in weight space (long-term NM), using the same approach as discussed in the first use case.

### 3.3. Use Case 3: Robot Locomotion Control and Online Adaptation

**Figure 10:**
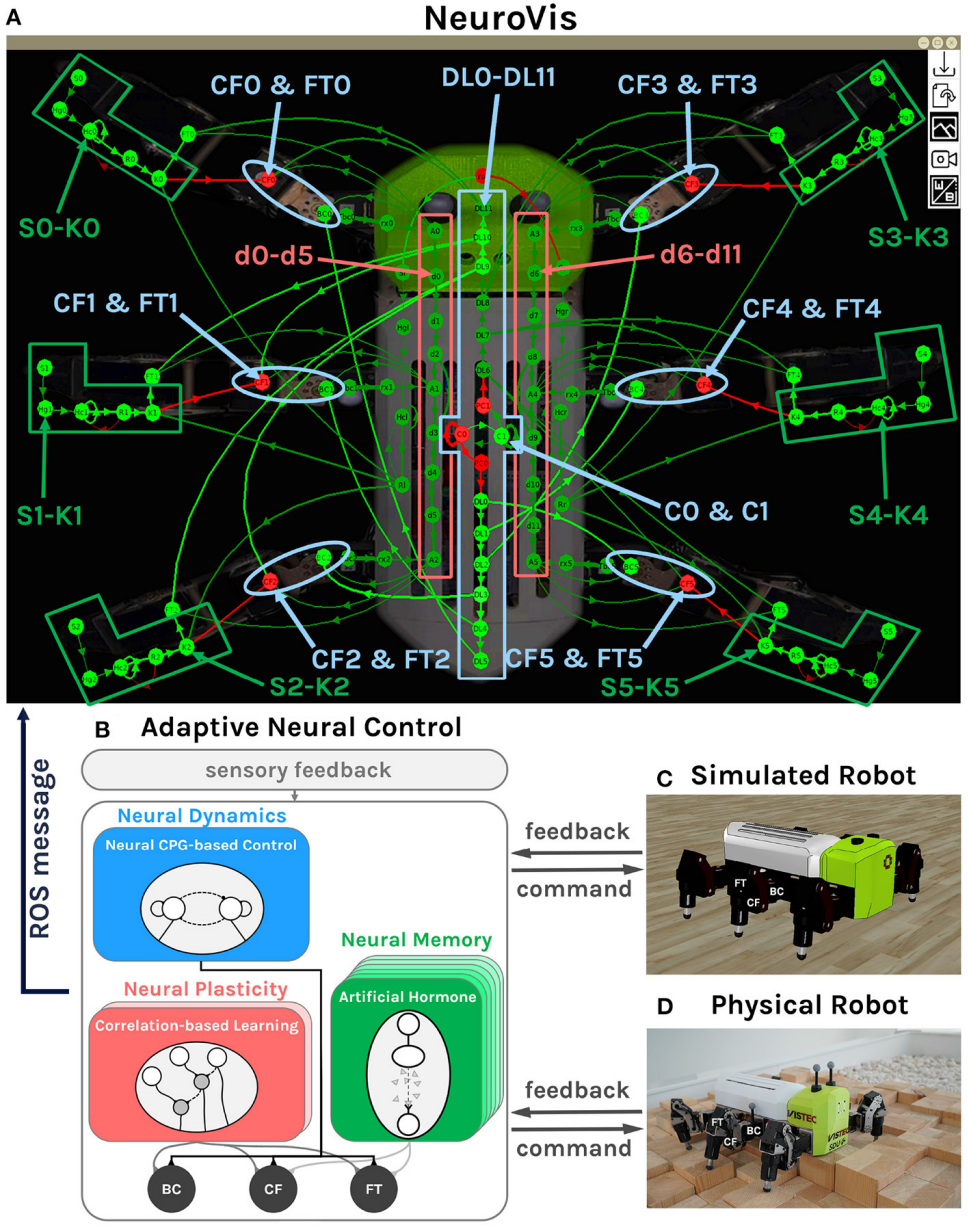
Neural visualization from NeuroVis showing the overview of the NS of the adaptive neural control network **(A)**. This neural visualization is created based on information published through ROS messages from the adaptive neural control. The ND is presented by the neurons C0, C1, DL0-DL11, CF0-CF5, and FT0-FT5 which are highlighted in blue. The NP is presented by the neurons d0-d11, which are highlighted in red. The NM is represented by the neurons S0-K0 to S5-K5, which are highlighted in green. **(B)** The diagram of the adaptive neural control with the highlighted neural components that are employed to present the ND, NP, and NM. **(C)** Simulated robot. **(D)** Physical robot (Thor and Manoonpong, 2019). A video of this use case can be seen at Supplementary Video 4^6^.

The final use case, presented in Supplementary Video 4^6^ and Figure 10, further demonstrates the NeuroVis-ROS interface and the interpretation of neural ingredients on a larger-scale neural network (Figure 10B). The neural network, consisting of 150 neurons with 200 connections, takes feedback (i.e., torque feedback) to generate adaptive locomotion pattern of a hexapod robot in order to cope with unseen environments. The network can demonstrate all neural ingredients: NS (all neurons and their connections), ND (highlighted in blue), NP (highlighted in red), and short-term NM (highlighted in green). This use case mainly presents the ability of NeuroVis to track and analyze (1) the ND during robot locomotion with different gaits, (2) the NP during the learning to step over an obstacle, and (3) the NM during leg adaptation to different terrains, underlying the control network. Here we aim to only show the performance of the visualization of NeuroVis rather than the control. Thus, the details of the control and its performance are not included here (see Homchanthanakul and Manoonpong, 2021 for more details).

Overall, the visualization operates as a python ROS node on a computer, receiving messages from another c++ ROS node of the neural network running on the robot. In this use case, NeuroVis can achieve a display rate of 40 frames per second. Apart from showing the NeuroVis-ROS interface, this use case presents an example of a general interpretation of complex neural control. Note that Supplementary Video 4^6^ is used in conjunction with the descriptions below.

#### 3.3.1. Neural Structure

The NS of the adaptive neural control network presented by NeuroVis is shown in Figure 10A and Supplementary Video 4^6^. The network consists of three main neural components with respect to the neural diagram shown in Figure 10B.

The first component, highlighted by the blue middle box (Figure 10A), includes C0, C1, and DL0-DL11 with feedforward and recurrent connections. It exploits the neural dynamics of a central pattern generator (C0 and C1) to generate basic rhythmic leg movement patterns for basic locomotion without sensory feedback (operating in an open-loop manner). The rhythmic patterns then propagate to the corresponding legs, highlighted by the blue ellipses, including CF0-CF5 and FT0-FT5 (Figure 10A). This neural component is used to present the ND.

The second component, highlighted by the red boxes (Figure 10A), includes d0-d11 with feedforward connections. It operates in a closed-loop manner with torque feedback for leg adaptation during a swing phase. It allows the robot to learn to proactively swing its middle and hind legs across an obstacle after the front leg hits it. The adaptation is done by a neural plasticity mechanism (see Manoonpong et al., 2013; Homchanthanakul and Manoonpong, 2021 for details). Thus, this component is used to present the NP and long-term NM.

The third component, highlighted by the green boxes (Figure 10A), includes S0-K0 to S5-K5 with feedforward and recurrent connections. It also operates in a closed-loop control manner with torque feedback for leg adaptation during a stance phase. It allows the robot to adapt its leg extension to walk on uneven terrain. The adaptation is done by artificial hormone mechanisms with embedded short-term neural memory (see Homchanthanakul et al., 2019; Homchanthanakul and Manoonpong, 2021 for details). This component is used to present the short-term NM.

#### 3.3.2. Neural Dynamics

According to Supplementary Video 4^6^, C0 and C1 (Figure 10A) blink alternately and generate a rhythmic pattern. The pattern then propagates through the delay line (DL6-DL11 in Figure 10A) and to the motor neurons that are responsible for leg lifting (CF0-CF5 and FT0-FT5). Between 0:38 and 0:46 min, the rhythmic pattern has a low frequency, resulting in a ripple appearing at DL6-DL11; thereby the robot uses a wave gait (one leg lifts at a time). Between 0:47 and 0:58 min, the pattern has a moderate frequency, leading to two ripples, so the robot uses a caterpillar gait (two legs lift at a time). Between 1:06 and 1:09 min, the pattern has a high frequency, causing three ripples, so the robot uses the tripod gait (three legs lift at a time).

#### 3.3.3. Neural Plasticity and Long-Term NM

With correlation-based neural learning (see Manoonpong et al., 2013; Homchanthanakul and Manoonpong, 2021 for details), the robot learns to predict the time that it should proactively swing its middle and hind legs across an obstacle after the front leg hits it. The robot learns to prolong the hitting signal from the front leg by increasing the connection weights (NP) between d6-d11 (Figure 10A). In Supplementary Video 4^6^ between 1:13 and 1:24 min, the signal from the front propagates too fast, so the mismatch activates the increasing of connection thickness. As the connections thicken, and the signal propagates slower. Finally, between 1:25 and 1:37 min, the signal reaches the middle and hind legs nearly at the same time when the middle and hind legs reach the obstacle. The connections remain the same afterward as the swing pattern is remembered through the connection weights.

#### 3.3.4. Short-Term NM

For online adaptation to deal with unexpected terrains (Homchanthanakul et al., 2019), the robot takes the difference between the expected foot contact signal and the real one as its input (i.e., S0–S5 in Figure 10A) and produces the signal controlling leg stretching (i.e., K0-K5 in Figure 10A). Thank to the short-term NM of this mechanism, the activities of K0-K5 are nearly the same even though the activities of S0-S5 abruptly changes for a short period as shown in Supplementary Video 4^6^. Between 1:42 and 1:47 min, the right front leg is on a rigid floor. S1 has a particular pattern; consequently, K1 activates at a certain level, and the leg stretches to such level. The activity of K1 is nearly the same even if S1 vanishes for a short period (see Supplementary Video 4^6^). Between 1:47 and 2:04 min, the robot is fully on a soft floor. The S1 pattern changes, resulting in a new K1 activity level and new stretching length.

## 4. Discussion and Conclusion

Although several existing approaches have been employed for presenting and analyzing the measured neural information of embodied neural systems, none of these has fully included the combined representations of the NS, ND, NP, and NM for real-time analysis (see Table 1 for the summary) (Pau and Johansen, 1990; Rusu et al., 2003; Capriglione et al., 2016; Cashman et al., 2017; Falotico et al., 2017; Lechner et al., 2019; Rudin, 2019; Chatzimparmpas et al., 2020; Mehmood et al., 2020; Simon, 2020; Tang et al., 2020). Therefore, NeuroVis is proposed as a tool/method to address this issue.

**Table 1:** Comparison of different methods, where ✔, ●, and ✘ denote inclusion, partial inclusion, and exclusion.

**Method**	**NS**	**ND**	**NP**	**NM**	**ROS**	**real-time**
● **XAI**						
***Post-hoc*** (Rudin, 2019)	✘	✘	✘	✘	✘	✘
**Feature importance** (Tang et al., 2020)	✘	●^*A*^	✘	✘	✘	✘
● **Information Analysis**						
**Connection matrix** (Capriglione et al., 2016)	✔	✘	✘	✘	✘	✘
**Cross-correlation** (Lechner et al., 2019)	✘	●^*A*^	✘	✘	✘	✘
**Mutual information** (Shwartz-Ziv and Tishby, 2017)	✘	●^*A*^	✘	✘	✘	✘
**Gradient** (Cashman et al., 2017)	✘	✘	✔	✘	✘	✘
**Weight analysis** (Pau and Johansen, 1990)	✘	✘	✘	●^*B*^	✘	✘
● **Visualization**						
**Graph** (Mehmood et al., 2020)	✘	●^*C*^	●^*C*^	●^*C*^	✘	✘
**Node-link diagram** (Rusu et al., 2003)	✔	●^*B*^	✘	●^*B*^	✘	✘
**Topography map** (Mehmood et al., 2020)	✔	●^*A*^	✘	✘	✘	✘
**Neurorobotics** (Falotico et al., 2017)	✔	●^*A*^	✘	✘	✔	✘
**Visualization** (Lechner et al., 2019)	✔	✔	✘	●^*B*^	✘	✔
**Brain simulator** (Simon, 2020)	✔	●^*C*^	●^*D*^	●^*D*^	✘	✔
**NeuroVis**	✔	✔	✔	✔	✔	✔

${●}^{A}$ : *It presents only the activity pattern without activity propagation*.

${●}^{B}$ : *It is a static representation*.

${●}^{C}$ : *It is suitable for a small amount of information*.

${●}^{D}$ : *It discretizes information into certain intervals (non-continuous)*.

NeuroVis translates the measured neural information to online NS, ND, NP, and NM visualization based on the degree of perceptiveness (Alexandre and Tavares, 2010). It firstly converts the connection matrix into a diagram of the NS. After that, information propagation, neural activities (ND), and weight change (NP) are projected onto the NS. Short-term NM is represented by retained neural activity due to the ND, while long-term NM is represented by converged connection thickness due to the NP.

In this work, the three use cases present the contribution and features of NeuroVis as follows:

(i) This method provides the visualization of all the spatial-temporal neural ingredients (i.e., NS, ND, NP, and NM) of various embodied neural systems with different network topologies and different applications (subsections 2.1, 3.1–3.3, Table 1).

(ii) Using solely NeuroVis, one can analyze all of the spatial-temporal neural ingredients. In contrast to analyzing the NS, ND, NP, and NM of an embodied neural system using node-link diagram, cross-correlation, and graph of connection weights and their update, respectively, all the analysis can be done using single visualization from NeuroVis (subsections 2.2, 3.1).

(iii) The proposed method can be used to simply explain and understand the overview and dynamics of neural systems in real-time by encoding neural activity and synaptic temporal dynamics into dynamic color gradients of connections and highlighting the change of connection weight with a luminous outline. As a result, the method facilitates the presentation of embodied neural systems by providing a more straightforward interpretation (subsection 2.3, subsection 3.1-3.3).

(iv) NeuroVis delivers real-time neural information measurement and visualization under the ROS framework. It can therefore visualize an embodied neural network in real-time (display freqeuncy ≥ 20*Hz*) while a robot is running (subsections 2.4, 3.2, 3.3).

**Figure 11:**
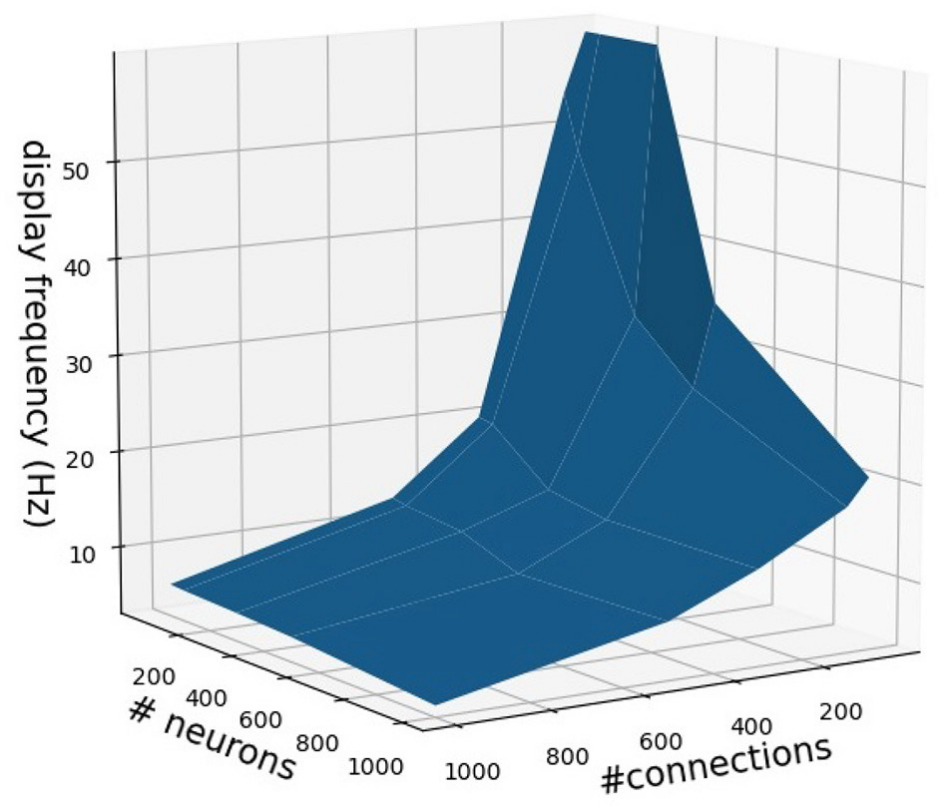
An approximation of NeuroVis's display frequency in relation to the number of neurons and connections. It should be noted that the approximation is based on NeuroVis running on a standard computer (Window 10, Intel® Core™ i7-8750H CPU @ 2.20 GHz 2.21 GHz, NVIDIA GeForce GTX 1050 GPU). The display frequency can be increased by using a computer with high computing power.

It is important to note that the NeuroVis real-time visualization is constrained by network size or complexity (i.e., number of neurons and connections) and computing power. In this study, NeuroVis achieves a display frequency of 40 Hz (or 40 frames per second) to visualize a neural control network, having a size of approximately 150 neurons and 200 connections, running on a standard computer (Window 10, Intel® Core™ i7-8750H CPU @ 2.20 GHz 2.21 GHz, NVIDIA GeForce GTX 1050 GPU). The display frequency can increase up to approximately 60 Hz with a smaller size neural network (< 50 neurons and < 200 connections). Increasing the number of neurons to 200 and connections to 400 reduces the display frequency to approximately 20 Hz. An approximation of NeuroVis's display frequency in relation to the number of neurons and connections is shown in Figure 11.

Taken altogether, NeuroVis is an alternative method for understanding and improving embodied neural mechanisms. It can be used to present novel neural systems and provide an explanation of such. For example, one can use NeuroVis to analyze and comprehend the underlying mechanisms of a neural system in order to 1) efficiently reduce/optimize its size, e.g., by removing unimportant (less active) neurons/connections (Han et al., 2015) and/or 2) efficiently scale it up by introducing new neural modules for new functions without destroying existing functions (Grinke et al., 2015; Thor et al., 2021). This will shape the way we build a neural system shifting from purely black box to white box or their combination toward explainable and understandable AI systems with trust and transparency (Loyola-Gonzalez, 2019).

Moreover, the potential of this method is not limited to non-spiking ANNs and 2D visualization. NeuroVis can also be applied to a spiking or hybrid network where the spiking activity information of a spiking neuron can be read and displayed as the neural activity information of a non-spiking neuron. We can visualize the (average) spiking activity and resting state of a spiking neuron through, e.g., green and red colors, respectively. However, to visualize the neural dynamics of a spiking or hybrid network properly, a computer with high computing power may be required to obtain a high display frequency. Besides, NeuroVis can be extended to 3D visualization with a hierarchical view of network activities and applied to other information visualization, e.g., the biological neural networks, brain models, and their signals (Falotico et al., 2017; Mehmood et al., 2020), where the brain structure can be the analogy of the NS, the activity/signal in a particular neuron/region could be modeled as the ND, and the relationship between the neurons and evolution of such relationship could be modeled as the NM and NP. When using NeuroVis for large-scale (biological or brain) network models, a higher level visualization (Mehmood et al., 2020) condensing the activity of groups of neurons and limiting the amount of information available will be required to aid in network behavior analysis. Accordingly, we will further investigate on applying mean-field and small-world network approaches (Dasgupta et al., 2011; Grabow et al., 2012; Gabrié, 2019; Kawamoto et al., 2019) to NeuroVis for implementing higher-level visualization. Last but not least, because of NeuroVis's real-time visualization capability, we can utilize it as a tool for developing and assessing embodied autonomous lifelong (continuous) learning and adaptation systems with robust and versatile behaviors (Homchanthanakul and Manoonpong, 2021; Logacjov et al., 2021).

## Data Availability Statement

The datasets presented in this study can be found in online repositories. The names of the repository/repositories and accession number(s) can be found below: https://gitlab.com/zumoarthicha/neurovis.git; www.manoonpong.com/NeuroVis/video1.mp4; www.manoonpong.com/NeuroVis/video2.mp4; www.manoonpong.com/NeuroVis/video3.mp4; www.manoonpong.com/NeuroVis/video4.mp4.

## Author Contributions

PM provided the general direction of the project, supervised the development of NeuroVis, and helped with data analysis and reviewed and edited the manuscript. AS developed NeuroVis, performed the experiments, and wrote the original manuscript. JH developed embodied neural control to validate NeuroVis and took part in robot experiments. All authors contributed to the article and approved the submitted version.

## Funding

This research was supported by the startup grant on Bio-inspired Robotics of Vidyasirimedhi Institute of Science and Technology (VISTEC).

## Conflict of Interest

The authors declare that the research was conducted in the absence of any commercial or financial relationships that could be construed as a potential conflict of interest.

## Publisher's Note

All claims expressed in this article are solely those of the authors and do not necessarily represent those of their affiliated organizations, or those of the publisher, the editors and the reviewers. Any product that may be evaluated in this article, or claim that may be made by its manufacturer, is not guaranteed or endorsed by the publisher.

## Footnotes

^1^NeuroVis software is publicly available at https://gitlab.com/zumoarthicha/neurovis.git.

^2^For example, in Manoonpong et al. (2007), a blue arrow represents an inhibitory synapse, while a red arrow represents an excitatory synapse, In Lechner et al. (2019), a red arrow represents an inhibitory synapse, while a green arrow represents an excitatory synapse.

^3^The video of the first use case is available at www.manoonpong.com/NeuroVis/video1.mp4.

^4^The video is available at www.manoonpong.com/NeuroVis/video2.mp4.

^5^The video of the second experiment is available at www.manoonpong.com/NeuroVis/video3.mp4.

^6^The video is available at www.manoonpong.com/NeuroVis/video4.mp4.

## References

[B1] Alexandre D. S. Tavares J. (2010). Introduction of human perception in visualization. Int. J. Imaging Rob. 4, 60–70. Availabe online at: https://www.researchgate.net/publication/277068044_Introduction_of_Human_Perception_in_Visualization/citations

[B2] Arena E. Arena P. Strauss R. Patané L. (2017). Motor-skill learning in an insect inspired neuro-computational control system. Front. Neurorobot. 11:12. 10.3389/fnbot.2017.00012 28337138 PMC5340754

[B3] Beer R. D. (2008). The dynamics of brain-body-environment systems: a status report, in Handbook of Cognitive Science, 99–120.

[B4] Briscoe M. H. (2012). Preparing Scientific Illustrations: A Guide to Better Posters, Presentations, and Publications. Springer Science & Business Media.

[B5] Capolei M. C. Andersen N. A. Lund H. H. Falotico E. Tolu S. (2020). A cerebellar internal models control architecture for online sensorimotor adaptation of a humanoid robot acting in a dynamic environment. IEEE Rob. Autom. Lett. 5, 80–87. 10.1109/LRA.2019.294381827295638

[B6] Capriglione D. Ferrigno L. Paciello V. Pietrosanto A. Vaccaro A. (2016). Experimental characterization of consensus protocol for decentralized smart grid metering. Measurement 77, 292–306. 10.1016/j.measurement.2015.09.024

[B7] Cashman D. Patterson G. Mosca A. Chang R. (2017). Rnnbow: visualizing learning via backpropagation gradients in recurrent neural networks, in Workshop on Visual Analytics for Deep Learning (VADL), Vol. 4.

[B8] Chatzimparmpas A. Martins R. M. Jusufi I. Kerren A. (2020). A survey of surveys on the use of visualization for interpreting machine learning models. Inf. Vis. 19, 207–233. 10.1177/1473871620904671

[B9] Dasgupta S. Manoonpong P. Woergoetter F. (2011). Small world topology of dynamic reservoir for effective solution of memory guided tasks. Front. Comput. Neurosci. 5:177. 10.3389/conf.fncom.2011.53.00177

[B10] Falotico E. Vannucci L. Ambrosano A. Albanese U. Ulbrich S. Vasquez Tieck J. C.. (2017). Connecting artificial brains to robots in a comprehensive simulation framework: the neurorobotics platform. Front. Neurorobot. 11:2. 10.3389/fnbot.2017.00002 28179882 PMC5263131

[B11] Gabrié M. (2019). Towards an understanding of neural networks: mean-field incursions (Ph.D. thesis). Paris Sciences et Lettres (ComUE).

[B12] Grabow C. Grosskinsky S. Timme M. (2012). Small-world network spectra in mean-field theory. Phys. Rev. Lett. 108, 218701. 10.1103/PhysRevLett.108.218701 23003310

[B13] Grinke E. Tetzlaff C. Wörgötter F. Manoonpong P. (2015). Synaptic plasticity in a recurrent neural network for versatile and adaptive behaviors of a walking robot. Front. Neurorobot. 9:11. 10.3389/fnbot.2015.00011 26528176 PMC4602151

[B14] Han S. Pool J. Tran J. Dally W. J. (2015). Learning both weights and connections for efficient neural networks. arXiv [preprint] arXiv:1506.02626.

[B15] Hohman F. Kahng M. Pienta R. Chau D. H. (2018). Visual analytics in deep learning: An interrogative survey for the next frontiers. IEEE Trans. Vis. Comput. Graph. 25, 2674–2693. 10.1109/TVCG.2018.2843369 29993551 PMC6703958

[B16] Homchanthanakul J. Manoonpong P. (2021). Continuous online adaptation of bioinspired adaptive neuroendocrine control for autonomous walking robots. IEEE Trans. Neural Netw. Learn. Syst. [Epub ahead of print]. 10.1109/TNNLS.2021.3119127 34669583

[B17] Homchanthanakul J. Ngamkajornwiwat P. Teerakittikul P. Manoonpong P. (2019). Neural control with an artificial hormone system for energy-efficient compliant terrain locomotion and adaptation of walking robots, in 2019 IEEE/RSJ International Conference on Intelligent Robots and Systems (IROS) (Macau: IEEE), 5475–5482.

[B18] Ijspeert A. J. Crespi A. Ryczko D. Cabelguen J.-M. (2007). From swimming to walking with a salamander robot driven by a spinal cord model. Science 315, 1416–1420. 10.1126/science.1138353 17347441

[B19] Kawamoto T. Tsubaki M. Obuchi T. (2019). Mean-field theory of graph neural networks in graph partitioning. J. Stat. Mech. 2019, 124007. 10.1088/1742-5468/ab3456

[B20] Knüsel J. Crespi A. Cabelguen J.-M. Ijspeert A. J. Ryczko D. (2020). Reproducing five motor behaviors in a salamander robot with virtual muscles and a distributed cpg controller regulated by drive signals and proprioceptive feedback. Front. Neurorobot. 14:604426. 10.3389/fnbot.2020.604426 33424576 PMC7786271

[B21] Koubâa A. (2019). Robot Operating System (ROS), Vol. 1. Springer.

[B22] Lechner M. Hasani R. Zimmer M. Henzinger T. A. Grosu R. (2019). Designing worm-inspired neural networks for interpretable robotic control, in 2019 International Conference on Robotics and Automation (ICRA) (Montreal, QC: IEEE), 87–94.

[B23] Logacjov A. Kerzel M. Wermter S. (2021). Learning then, learning now, and every second in between: lifelong learning with a simulated humanoid robot. Front. Neurorob. 78:669534. 10.3389/fnbot.2021.669534 34276332 PMC8281815

[B24] Loyola-Gonzalez O. (2019). Black-box vs. white-box: understanding their advantages and weaknesses from a practical point of view. IEEE Access 7, 154096–154113. 10.1109/ACCESS.2019.294928627295638

[B25] Luque N. R. Garrido J. A. Carrillo R. R. D'Angelo E. Ros E. (2014). Fast convergence of learning requires plasticity between inferior olive and deep cerebellar nuclei in a manipulation task: a closed-loop robotic simulation. Front. Comput. Neurosci. 8:97. 10.3389/fncom.2014.00097 25177290 PMC4133770

[B26] Manoonpong P. Kolodziejski C. Wörgötter F. Morimoto J. (2013). Combining correlation-based and reward-based learning in neural control for policy improvement. Adv. Complex Syst. 16:1350015. 10.1142/S021952591350015X

[B27] Manoonpong P. Pasemann F. Roth H. (2007). Modular reactive neurocontrol for biologically inspired walking machines. Int. J. Rob. Res. 26, 301–331. 10.1177/0278364906076263

[B28] Mehmood R. M. Yang H.-J. Kim S.-H. (2020). Children emotion regulation: development of neural marker by investigating human brain signals. IEEE Trans. Instrum. Meas. 70, 1–11. 10.1109/TIM.2020.301181733776080

[B29] Ming Y. Cao S. Zhang R. Li Z. Chen Y. Song Y.. (2017). Understanding hidden memories of recurrent neural networks, in 2017 IEEE Conference on Visual Analytics Science and Technology (VAST) (Phoenix, AZ: IEEE), 13–24.

[B30] Nordlie E. Plesser H. E. (2010). Visualizing neuronal network connectivity with connectivity pattern tables. Front. Neuroinform. 3:39. 10.3389/neuro.11.039.2009 20140265 PMC2816167

[B31] Pasemann F. Hild M. Zahedi K. (2003). So (2)-networks as neural oscillators, in International Work-Conference on Artificial Neural Networks (Berlin; Heidelberg: Springer), 144–151. Available online at: http://www.neurorobotik.de/downloads/publications/2003%20Pasemann%20-%20SO(2)-Networks%20as%20Neural%20Oscillators.pdf.

[B32] Pau L. Johansen F. (1990). Neural network signal understanding for instrumentation. IEEE Trans. Instrum. Meas. 39, 558–564. 10.1109/19.5723327295638

[B33] Ribeiro M. T. Singh S. Guestrin C. (2016). “why should i trust you?” explaining the predictions of any classifier, in Proceedings of the 22nd ACM SIGKDD International Conference on Knowledge Discovery and Data Mining, 1135–1144.

[B34] Rudin C. (2019). Stop explaining black box machine learning models for high stakes decisions and use interpretable models instead. Nat. Mach. Intell. 1, 206–215. 10.1038/s42256-019-0048-x PMC9122117 35603010

[B35] Rusu P. Petriu E. M. Whalen T. E. Cornell A. Spoelder H. J. (2003). Behavior-based neuro-fuzzy controller for mobile robot navigation. IEEE Trans. Instrum. Meas. 52, 1335–1340. 10.1109/TIM.2003.81684627295638

[B36] Schilling M. Cruse H. (2020). Decentralized control of insect walking: a simple neural network explains a wide range of behavioral and neurophysiological results. PLoS Comput. Biol. 16:e1007804. 10.1371/journal.pcbi.1007804 32339162 PMC7205325

[B37] Schilling M. Paskarbeit J. Hoinville T. Hüffmeier A. Schneider A. Schmitz J.. (2013). A hexapod walker using a heterarchical architecture for action selection. Front. Comput. Neurosci. 7:126. 10.3389/fncom.2013.00126 24062682 PMC3774992

[B38] Senk J. Carde C. Hagen E. Kuhlen T. W. Diesmann M. Weyers B. (2018). Viola–a multi-purpose and web-based visualization tool for neuronal-network simulation output. Front. Neuroinform. 12:75. 10.3389/fninf.2018.00075 30467469 PMC6236002

[B39] Shaikh D. Manoonpong P. (2019). A neuroplasticity-inspired neural circuit for acoustic navigation with obstacle avoidance that learns smooth motion paths. Neural Comput. Appl. 31, 1765–1781. 10.1007/s00521-018-3845-y

[B40] Sheh R. (2017). “why did you do that?” explainable intelligent robots, in AAAI Workshop-Technical Report, 628–634.

[B41] Shwartz-Ziv R. Tishby N. (2017). Opening the black box of deep neural networks via information. arXiv [Preprint] arXiv:1703.00810.

[B42] Simon C. J. (2020). New brain simulator II open-source software, in International Conference on Artificial General Intelligence (Cham: Springer), 317–321.

[B43] Srisuchinnawong A. Wang B. Shao D. Ngamkajornwiwat P. Dai Z. Ji A.. (2021). Modular neural control for gait adaptation and obstacle avoidance of a tailless gecko robot. J. Intell. Rob. Syst. 101:27. 10.1007/s10846-020-01285-y

[B44] Sutton R. S. McAllester D. A. Singh S. P. Mansour Y.. (1999). Policy gradient methods for reinforcement learning with function approximation, in NIPs, Vol. 99 (Citeseer), 1057–1063.

[B45] Szadkowski R. Prágr M. Faigl J. (2021). Self-learning event mistiming detector based on central pattern generator. Front. Neurorobot. 15:9652. 10.3389/fnbot.2021.629652 33613224 PMC7890245

[B46] Tang H. Liao Z. Chen P. Zuo D. Yi S. (2020). A novel convolutional neural network for low-speed structural fault diagnosis under different operating condition and its understanding via visualization. IEEE Trans. Instrum. Meas. 70:3501611 10.1109/TIM.2020.301675227295638

[B47] Thor M. Kulvicius T. Manoonpong P. (2020). Generic neural locomotion control framework for legged robots. IEEE Trans. Neural Netw. Learn. Syst. 32, 4013–4025. 10.1109/TNNLS.2020.3016523 32833657

[B48] Thor M. Manoonpong P. (2019). A fast online frequency adaptation mechanism for cpg-based robot motion control. IEEE Rob. Autom. Lett. 4, 3324–3331. 10.1109/LRA.2019.292666031395565

[B49] Thor M. Strohmer B. Manoonpong P. (2021). Locomotion Control With Frequency and Motor Pattern Adaptations. Front. Neural Circuits 15:743888. 10.3389/fncir.2021.743888 34899196 PMC8655109

